# Do nutrition and cash-based interventions and policies aimed at reducing stunting have an impact on economic development of low-and-middle-income countries? A systematic review

**DOI:** 10.1186/s12889-019-7677-1

**Published:** 2019-10-30

**Authors:** Nathaly Aguilera Vasquez, Jana Daher

**Affiliations:** SMART initiative at Action Against Hunger Canada, 500-720 Bathurst St., Toronto, Ontario ON M5S 2R4 Canada

**Keywords:** Childhood stunting, Childhood mortality, Human capital, Economic development, Low-and-middle-income countries

## Abstract

**Background:**

Childhood stunting is the most common manifestation of chronic malnutrition. A growing body of literature indicates that stunting can have negative repercussions on physical and cognitive development. There are increasing concerns that low- and middle-income countries (LMICs) are particularly susceptible to adverse consequences of stunting on economic development. The aim of this review is to synthesize current evidence on interventions and policies that have had success in reducing stunting and explore the impact of successes on economic indicators.

**Methods:**

This review adheres to the Preferred Reporting Items for Systematic Reviews and Meta-Analyses guidelines. Articles were searched through MEDLINE via PubMed and Ovid, Cochrane Library, Web of Science and ProQuest. Only articles that addressed the effects of nutrition and cash-based interventions and/or policies on stunting and reported effects on childhood mortality and/or human capital indicators were included. Two reviewers independently abstracted data and assessed quality.

**Results:**

Seventeen studies from Africa (47%), South America (41%), and South Asia (12%) met the eligibility criteria: 8 cohort studies, 4 case studies, 4 Randomized Control Trials (RCTs) and 1 quasi-trial. Three types of interventions/policies were evaluated: multisectoral policies, nutritional supplementations and cash-based interventions (CCT). Overall, 76% of the included studies were successful in reducing stunting and 65% of interventions/policies reported successes on stunting reductions and economic successes. Five of the 11 successful studies reported on nutritional supplementation, 4 reported on multisectoral policies, and 2 reported on CCT interventions. Average Annual Rate of Reduction (AARR) was calculated to assess the impact of multisectoral policies on childhood mortality. AARR for under 5 mortality ranged from 5.2 to 6.2% and all countries aligned with the global target of 4.4% AARR. Quality assessment yielded positive results, with the biggest concerns being attrition bias for cohort studies, blinding for trials and generalizability of results for case studies.

**Conclusions:**

Evidence suggests that investment in fighting chronic malnutrition through multisectoral policies, multi-year nutritional supplementation (protein or multiple micronutrient supplementation) and possibly CCTs can have a long-term impact on economic development of LMICs. More evidence is needed to inform practices in non-represented regions while prioritizing standardization of economic indicators in the literature.

## Background

The term stunting refers to linear growth retardation and decreased development in children [1] caused by chronic malnutrition. According to WHO standards, a child is stunted if their height-for-age (HAZ) is at least two standard deviations below the WHO Child Growth Standards median [2]. Many factors pave the way for a child to become stunted, particularly if these factors are present in the first 1000 days following conception [3], a crucial period where most stunting occurs [3] and a critical time for the cognitive development of a child [4]. Generally, stunting is caused by poor maternal health and nutrition during pregnancy, recurrent childhood infection (particularly diarrheal infection) and inappropriate Infant and Young Child Feeding (IYCF) practices mainly due to inadequate breastfeeding and complementary feeding [3]. Other important factors that may lead to stunting are fetal growth restriction, preterm birth and small for gestational age [5]. Furthermore, maternal characteristics such as teenage motherhood, short birth intervals [5] and short stature [6] are also associated with stunting, as well as environmental factors such as improper sanitation practices [5] and food insecurity [7]. The first 1000 days is a critical window for brain development, and a lack of proper nutrient intake in this period can hinder development, and will have disastrous consequences on the cognitive capacity of a child [4]. Although the definition of stunting is limited to its physical consequences, its effect on brain maturation cannot be overlooked.

In 2018, stunting was estimated to affect 21.9% of children under 5 years of age globally which equates to 149 million children worldwide that are not reaching their full developmental potential [8]. The overwhelmingly increasing body of evidence that points to stunting as the source of important human capital loss has put stunting at the forefront of health priorities that must be addressed by the international community [2, 9–11]. In 2012, the World Health Assembly (WHA) created 6 global nutrition targets to be achieved by 2025, the first of which is the reduction of stunting amongst children under 5 by 40% [2]. Furthermore, stunting is a global issue that disproportionately affects low- income and lower-middle-income countries (LMICs) where 39.6 and 96.8 million children, respectively, struggle with this disorder [8]. In comparison, 2.1 million children are affected in high-income countries [8].

Stunting is distinguished from other nutritional disorders in that it is irreversible if not addressed within the first 1000 days. Although there is evidence of catch-up growth in stunted children during adolescence [12], the same does not apply to brain development. This is why the only way to address stunting is to prevent it. Stunting in early childhood leads to disastrous long-term consequences that affect the child’s ability to fulfill roles in personal, social and economic spheres [13]. The WHA compiled a set of recommendations that, if put to action, are expected to prevent stunting and catalyze the achievement of the stunting goal for 2025 [2]. These recommendations focus on the improvement of assessment techniques and understanding of stunting, as well as the scaling-up of coverage of evidence-based interventions aimed at improving maternal and child health and promoting exclusive breastfeeding and appropriate complementary feeding practices. They also highlight the importance of community-based interventions.

In this paper, we performed a systematic review of evidence from the past decade (2008–2019) investigating how policy and interventions aimed at reducing stunting can bring about economic development in low- and middle-income countries (LMICs) by observing their impact on human capital indicators and childhood mortality. The authors decided to restrict the study to LMICs because children living in LMICs are primarily affected by stunting, and there is a need to understand how to decrease prevalence and address consequences of stunting in these countries. Previous reviews have investigated the long-term consequences of stunting on human capital, as well as other long-term consequences of stunting [9, 13–15] and the relationship between stunting and economic outcomes [10]. However, these reviews did not evaluate specific programs or interventions that may have had an impact on stunting reduction. Further reviews have evaluated the impact of programmes on stunting reduction [16], but did not make a link with economic outcomes. To our knowledge no other review has explored the impact of policies and nutrition and cash-based interventions aimed at reducing stunting on a range of economic indicators.

### Rationale for using human capital indicators and childhood mortality as measures of economic loss

The Organization for Economic Co-operation and Development (OECD) defines human capital as the “knowledge, skills, competencies and attributes embodied in individuals that facilitate the creation of personal, social and economic well-being” [17]. Human capital is pivotal in increasing economic productivity of adults [17]. Previously published reviews have supported the link between stunting and cognitive, educational and economic outcomes later in life [14, 15]. For example, adults who were stunted as children have been shown to have lower wages [15]. Furthermore, Hoddinott et al. hypothesized the pathways through which stunting in early years of life may lead to economic losses in adulthood [9]. The pathway identifies three contributing factors to economic loss: decreased stature, low cognitive development (i.e. poor grade attainment, loss of locomotor skill potential, diminished attention and memory capacity), and a high risk of developing chronic diseases. All of these factors represent important losses to the economy of a country.

According to UNICEF data, half of all deaths of children under 5 can be attributed to undernutrition [18]. Furthermore, stunting is the most prevalent nutritional disorder caused by undernutrition [19]. A recent pooled analysis of 10 prospective studies showed that children with severe stunting (HAZ < − 3 SD ) had a 5.48 times higher hazard of dying when compared to children who had a HAZ score ≥ − 1 [20]. This same study also established that among the 10 studies analyzed, undernutrition led to mortality mainly through infection (primarily respiratory tract infection) and diarrhea.

Furthermore, childhood mortality not only represents the inability of governments to provide children with access to proper resources to ensure good health, but it is also a major threat to the economy. A recent study that assessed the effect of childhood mortality on non-health GDP (Gross Domestic Product) loss in the African region, found that the 2.976 million child deaths that occurred in this region in 2013 could lead to a total loss of over 150 billion dollars in future non-health GDP (cumulative amount for all the countries in the African region), with a higher burden affecting lower-middle-income countries in this region [21]. With such a significant estimated loss in non-health GDP in this region, it becomes apparent that childhood mortality is an integral part of economic growth. Thus, it is important to also consider childhood mortality and morbidity, and not only human capital, when exploring economic loss and stunting.

This review will consider two broad categories of economic indicators: human capital indicators and childhood mortality. The diverse terminology used in the literature to refer to each indicator is described in Additional file 1.

## Methods

We aimed to conduct a systematic review of the literature to synthesize evidence from the past decade investigating nutrition and cash-based interventions and policies that have had success in reducing stunting and explore the impact of those successes on economic indicators. The PRISMA (Preferred Reporting Items for Systematic Reviews and Meta-Analyses) guidelines were followed. We searched MEDLINE via PubMed, Cochrane Library, MEDLINE via Ovid, Web of Science and ProQuest for a 10-year period from 2008 to 2018. The search was initially conducted on June 4th, 2018 and updated on July 12th, 2019.

### Search strategy

We used keywords to search the 5 databases and the strategy was adapted to the specific requirements of each. Keywords related to “nutrition policy or intervention” AND “stunting” AND “economic” AND “low-and middle-income countries” were identified. The search strategy adapted to PubMed is presented in Additional file 2: Table S2.

### Study selection and eligibility criteria

**Fig. 1:**
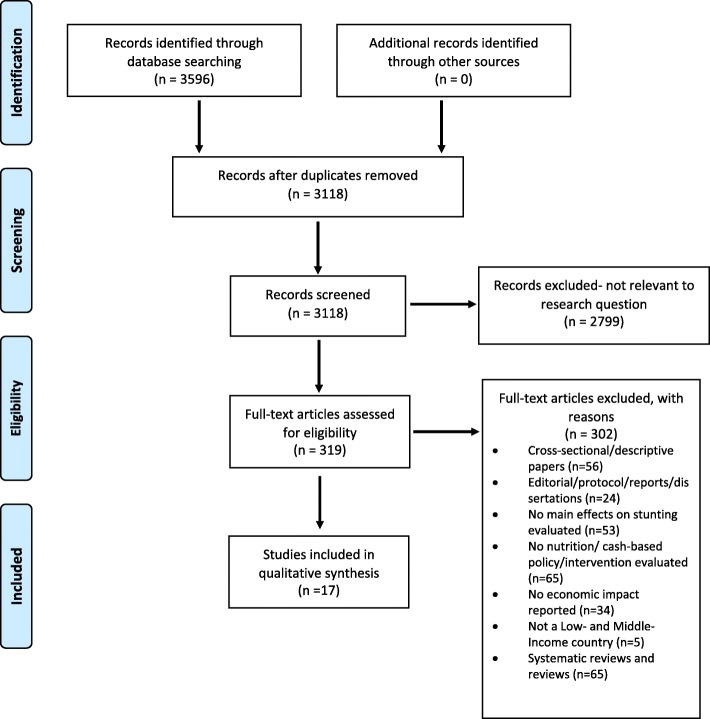
PRISMA flow chart

Articles were screened and evaluated for eligibility. Any study in the English language (case study, cohort study, Randomized Control Trial (RCT)) that addressed the effects of nutrition and cash-based interventions and/or policies on stunting and reported effects on economic indicators (cognitive health, economic outcomes, chronic disease, childhood mortality) was included in the study. Only studies where participants were exposed to an intervention or policy under 10 years of age were included. A grey literature search was conducted; however, no relevant literature was identified. The authors ensured the inclusion of articles reporting on both positive and negative results in order to limit publication bias in the study. Studies were excluded on the basis of study design and/or outcomes of interest and/or the location of the study. See Fig. 1 for the full breakdown of the article selection process.

### Data abstraction and quality assessment

Two reviewers independently screened the articles for eligibility, abstracted data from the included articles and assessed the quality of the selected articles (JD and NA). Any disagreement was discussed and resolved among the reviewers. A data abstraction table was used to report on the following categories: authors, country, study design, study population and sample size, policy/intervention type, length of the intervention/policy, stunting reduction (yes/no), impact on stunting, economic indicators assessed and impact on economic indicators. To assess the quality of the studies the reviewers used: the Newcastle-Ottawa Scale for cohort studies, the Cochrane Risk of Bias tool for trials (RCTs and quasi-trials) and the CEMBa (Center for Evidence-Based Management) tool [22] for case studies.

### Impact assessment and presentation of results

For cohort studies and trials, impact was assessed based on statistical significance of the results of each study on stunting and specific economic indicators. For case studies, impact on childhood mortality was evaluated by calculating the Average Annual Rate of Reduction (AARR) using a methodology previously outlined by UNICEF [23]. This allowed for the unbiased comparison of childhood mortality reductions between case studies, as well as with the global target of 4.4% AARR set for the period of 1990 to 2015 [24]. In order to achieve this calculation, data on country prevalence of Under 5 Mortality Rate (U5MR) was gathered based on available information in the WorldBank database for the period of 2000–2017 for all countries evaluated in the case studies [25] (Additional file 3: Tables S3-S6). Results were presented by first outlining studies that were not successful in reducing stunting, and then differentiating by intervention/policy type those studies that were successful in reducing stunting and describing their impact on economic indicators.

## Results

### Descriptive summary of studies

Out of 2782 articles obtained through the 5 different databases searched, 319 were retained following initial screening and assessed for eligibility. Ultimately, 17 of the 319 articles (5.3%) met the eligibility criteria and were included in the analysis (Fig. 1). Results from the data abstraction process are presented in Table 1. The analysis included studies located in 10 different LMICs. 8 of the 17 studies were conducted in sub-Saharan Africa (3 in Ethiopia [26–28], 2 in Malawi [29, 30], 1 in Gambia [31], 1 in Niger [32] and 1 in Zambia [33]), 7 of the 17 studies were implemented in South America (3 in Guatemala [34–36], 2 in Peru [37, 38] and 2 in Mexico [39, 40]), and 2 of the 17 were implemented in South Asia (1 in India [41] and 1 in Pakistan [42]). Overall, 8 cohort studies, 4 RCTs, 4 case studies and 1 quasi-experimental study were included in this review. The 4 case studies focused on various national multisectoral government policies. Of the cohort studies, 62.5% reported outcomes of a nutritional supplementation and 37.5% reported on conditional cash transfers (CCT). Of the RCTs, 50% reported on a multiple micronutrient supplementation and 50% involved a single micronutrient supplementation. The quasi-experimental study involved a complementary feeding intervention paired with micronutrient supplementation. Overall, 10 studies evaluated nutrition interventions, 4 reported on multi-sectoral policies, and 3 reported on CCT.

**Table 1:** Data abstraction table

Author and Year	Country	Study Design	Study Population and Sample Size	Policy/intervention type	Length of intervention/ policy	Stunting reduction (yes/no)	Impact on stunting	Economic indicators	Impact on economic indicators
Amouzou, A. et al., 2012 [32]	Niger	Case Study	Children under 5 in Niger *n* = N/A	Multisectoral programmes and policies focused on maternal, newborn and child health	On-going since 1996	Yes	Decrease in stunting prevalence for children aged 24–35 months from 67 to 54% in 2009 (13% reduction)	Childhood mortality	5.1% annual reduction in U5MR with 10% of this reduction attributed to reductions in stunting prevalence.
Huicho, L. et al., 2016 [37]	Peru		Children under 5 in Peru *n* = N/A	Policies and programmes for reducing poverty, reforming the health sector, and improving reproductive health, nutrition and maternal and child health.	On-going since 2005	Yes	Stunting prevalence decreased from 30% in 2000 to 17.5% in 2013 Calculate % reduction		Under 5 mortality decreased by 58% (from 2000 to 2013).
Kanyuka, M. et al., 2016 [29]	Malawi		Children under 5 in Malawin = N/A	Policies, programmes and funding allocation that aimed to increase coverage of high-impact interventions that addressed the main causes of childhood mortality	On-going since 1990’s	Yes	Stunting deceased by 11.1% between 2000 and 2013		Under 5 mortality fell from 247 deaths/1000 livebirths to 71/1000 from 1990 to 2013 with 8.6% of this reduction attributed to reductions in stunting prevalence.
Ruducha, J., 2017 [26]	Ethiopia		Children under 5 in Ethiopia *n* = N/A	Policies and programmes for reducing poverty and improving agricultural productivity, food security, water supply, and sanitation.	On-going since before 2000’s	Yes	Stunting prevalence decreased from almost 60% in 2000 to 40% in 2014 (20% decrease)		Under 5 mortality decreased from 205 deaths/1000 livebirths in 1990 to 64 deaths/1000 livebirths in 2013 with 44% for this reduction attributed to reductions in stunting prevalence.
Andersen, T. et al., 2015 [38]	Peru	Prospective cohort study	Children 7–8 in Peru *n* = 1960	Conditional Cash Transfer programme (CCT) “Juntos”	On-going since 2005	Yes	Only in boys exposed to CCT for 2y or more: 0.43-point increase in HAZ score [95% CI: 0.09–0.77], *p* < 0.01)	Language development and school achievement	No significant effects observed.
Fernald, L.C.H. et al., 2008 [39]	Mexico	Retrospective cohort study	Children 24–68 months old who were exposed to “Opportunidades” their whole lives *n* = 2449 children	Conditional Cash Transfer programme “Opportunidades”	On-going since 1998	Yes	Doubled cash transfer associated with: 0.2 [95%CI: 0.09–0.30], *p* < 0.0001) increase in HAZ score, 10% 0(*p* < 0.0001) decrease in stunting	Cognitive, motor and language development	Doubled cash transfer associated with: 0.06 (p = 0.67) points higher in motor skill and 1.15 (*p* = 0.001) higher in endurance; 0.12 (*p* = 0.002) point higher in long-term memory, 0.13 (*p* < 0.0001) points higher in short-term memory and 0.08 (*p* = 0.02) point higher in visual integration;: 0.18 (*p* < 0.0001) higher language score
Fernald, L.C.H. et al., 2009 [40]	Mexico	Retrospective cohort study	Children 8–10 years who were part of an early vs. late (18 mo. difference) introduction to Conditional Cash Transfer programme “Opportunidades” *n* = 1793	Conditional Cash Transfer programme “Opportunidades”	On-going since 1998	Yes	Additional 18 mo. Of CCT for children of uneducated mothers: 1.5 cm (*p* = 0.029) increase in HAZ/ 0.23-point HAZ increase (95% CI = 0.23–0.44)	Cognitive, language and socioemotional development	Early vs. late treatment: Reduced behavioural problems (mean score − 0·09 vs. 1·03 (*p* = 0·0024)
Hoddinnott, J. et al., 2008	Guatemala	Retrospective Cohort Study	Adults 25–42 exposed to supplementation trial from ages 0 to 7 *n* = 1338	nutritional supplementation (protein supplementation beverage “atole”)	8 years	Yes	- Stunting to < 20% (from baseline of 45%) -2.4 cm (*p* < 0.005) increase in length of children at 3 years of age	Annual income, hours worked, average hourly wages	For exposure at 0–24 months only: 0.665 US$/h (*p* = 0.009) higher
Hoddinott, J. et al., 2013	Guatemala	Retrospective cohort study	Adults 25–42 exposed to supplementation trial from ages 0 to 7 *n* = 1338	Nutritional supplementation (protein supplementation beverage “atole”)	8 years	Yes	- Stunting to < 20% (from baseline of 45%)-2.4 cm (p < 0.005) increase in length of children at 3 years of age	Schooling-related outcomes, health outcomes, labor market outcomes	1 SD improvement in HAZ leads to: 0.78 (*p* = 0.003) increase in highest grade attained, 0.28 (*p* = 0.003) increase in reading score, 0.25 increase in non-verbal cognitive ability (0.002); 5 (*p* = 0.003) increase in hand strengthBeing stunted at 24 mo. leads to: leaving school 3.14 (*p* = 0.026) years younger, Achieving 4.64 (*p* = 0.004) less school grades, 1.26 (0.013) lower reading score, 1.12 (*p* = 0.006) lower nonverbal cognitive ability score, 22 point (p = 0.008) lower hand strength, 41% (0.017) lower likelihood of being skilled laborer or white collar worker
Kinra, S. et al., 2008 [41]	India	Retrospective cohort study	13–18 year-old adolescents who were part of nutrition and public health intervention as children (under 6 years old) *n* = 1165 adolescents	Nutritional supplementation (protein-calorie supplementation) and public health programmes	3 years	Yes	In exposed to intervention: 14 mm (*p* = 0.07) taller	Cardiovascular health	20% (*p* = 0.02) more favorable insulin resistance index, 3.3% (P = 0.008) more favorable arterial health
Nkhoma, O.W.W. et al., 2013 [30]	Malawi	Prospective cohort study	6–8-year-old children in Malawi *n* = 226 children	School feeding program (increased micronutrient and caloric intake)	1 year	No	No significant effects on linear growth in comparison to control school	Cognitive ability	After 9-month exposure to supplement: decrease in errors made in one element of learning from 24.9 in control to 18.5 in intervention (p-interaction = 0.02)
Stein, A.D. et al., 2008 [36]	Guatemala	Prospective cohort study	Adults 25–42 exposed to supplementation trial from ages 0 to 7 *n* = 1448	Nutritional supplementation (protein supplementation beverage “atole”)	8 years	Yes	- Stunting to < 20% (from baseline of 45%) -2.4 cm (p < 0.005) increase in length of children at 3 years of age	Reading comprehension, cognitive functioning	Exposure to supplement 0–12 mo.:6.39 [95%CI: 0.79–11.99] increase in reading comprehension score, 2.09 [95%CI: 0.79–3.99] increase in cognitive functioning score.
Argaw, A. et al., 2018 [27]	Ethiopia	2 × 2 factorial randomized controlled trial	Mothers and their children ages 6–12 months in 3 districts of the Jimma zone in Southwest Ethiopia between November 2013 to February 2015 *n* = 360 mother-infant pairs	Nutritional supplementation (n-3 long-chain PUFA supplementation)	12 months	No	No significant effect of the supplementation was observed on linear growth.	Child health	No significant effect on morbidity or systemic inflammation.
van der Merwe, L.F. et al., 2013 [31]	Gambia	Randomized, double-blind control trial	Rural Gambian infants (3–9 months old) *n* = 172	Nutritional supplementation (long-chain PUFA supplementation)	6 months	No	No significant effect of the supplementation was observed on linear growth.	Cognitive development and morbidity in the infants	No significant effects on cognitive development, intestinal function or morbidity were detected.
Yousafzai, A.K. et al., 2014 [42]	Pakistan	Cluster-randomised 2X2 factorial effectiveness trial	Children 0–24 months old *n* = 1489	Nutritional supplementation (multiple micronutrient powders)	3 years	Yes	Nutritional supplementation resulted in: 0.2 increase in HAZ score (p < 0.0001) at 6 mo. and 0.2 increase in HAZ score (*p* = 0.02) at 18 months.	Cognitive, language, motor and social-emotional development and child health	Nutritional supplementation resulted in: 3.4 higher cognition scores [95%CI: 1.1–5.7], 5.1 higher language scores [95%CI: 2.9–7.3], 2.9 higher motor score [95%CI: 0.5–5.3].
Samuel, A. et al., 2018 [28]	Ethiopia	Quasi-experimental matched-control study	Children 6–23 months old *N* = 1172	Nutritional supplementation (complementary feeding program paired with low iron dose multiple micronutrient powders)	37 weeks	Yes	Nutritional supplementation resulted in: 0.18 (SE: 0.05, *p* < 0.05) significant increase in HAZ score and an odds ratio (OR) for stunting of 0.49 [95% CI: 0.40–0.60] after 37 weeks of intervention (i.e. 51% reduced odds of stunting in the intervention group)	Child health	No significant positive effects observed on child health.
Masuda, K. & Chitundu, M., 2019 [33]	Zambia	Two-arm randomized control trial	Children 6–18 months of age *N* = 547	Nutritional supplementation (Micronutrient supplementation using spirulina platensis)	12 months	No	No significant effect on linear growth.	Child health and motor development	Nutritional supplementation resulted in: Reduction in the incidence of cough by 11% [95% CI : -0.23–0.00] and non-significant reductions in incidence of pneumonia (−0.17, [95% CI: −0.17,0.04]), severe fever (− 0.03, 95% CI = − 0.13, 0.06) and fever (− 0.09, [95% CI: − 0.19, 0.02]) Increase in probability of being able to walk alone by 15 months by 8% [95%CI : 0.02–0.14]

Furthermore, the age range of participants in the included studies varied. 5 of the 17 studies studied effects of interventions on children aged 0–24 months, 4 of the 17 studies focused on children under 5 years of age, 4 of the 17 studies reported on effects in slightly older children (6–10 years), and 4 of the 17 studies examined longer term outcomes in adolescents aged 13–18 years old and in adults aged 25–42 years. Sample sizes across all studies ranged from 172 to 2449. The sample sizes for the case studies were not reported because analyses for these studies were done using secondary data gathered from National surveys. Economic indicators examined across the studies were classified into two broad categories: human capital indicators and childhood mortality indicators (see Additional file 1: Table S1). 8 of the 17 studies focused solely on human capital indicators, 6 of the 17 studies focused solely on childhood mortality indicators and 3 of the 17 studies reported results on both human capital and childhood mortality indicators.

Overall, 11 of the 17 included studies (65%) reported positive effects on both stunting reduction and on the economic indicators assessed. 5 of the 11 successful articles reported on nutritional supplementation [34–36, 42, 43], 4 reported on multisectoral policies [26, 29, 32, 37], and 2 reported on CCT [39, 40].

#### Studies without demonstrated effects on stunting reduction

4 out of 17 of the included studies did not demonstrate positive effects on stunting reduction, and all were nutritional supplementation studies. This includes a 1- year cohort study of a school feeding program in Malawi aimed at 6 to 8-year-old children [30]. Also unsuccessful were 2 RCTs involving an oil-based, single micronutrient nutritional supplementation, conducted in Gambia and Ethiopia which lasted 6 and 12 months respectively and were both directed at children aged below 24 months [27, 31]. Furthermore, a 12-month multiple micronutrient supplementation RCT implemented in Zambia was not successful in enhancing physical growth of infants aged 6–18 months [33].

#### Economic impact of nutritional interventions successful in reducing stunting

Ten of the 17 included articles reported on nutritional supplementation interventions, but only 6 of the 10 were successful in reducing stunting. Overall, nutritional supplementations were implemented for periods of 6 months to 7 years across all studies. Indicators of interest in nutritional supplementation studies were both childhood mortality and human capital indicators.

In three studies [34–36], the authors investigated adult outcomes of a nutrition supplementation trial that took place in Guatemala between 1969 and 1977. This intervention involved a protein supplementation beverage named *atole*. This trial resulted in approximately a 25% decrease in stunting prevalence in the intervention sites (from a baseline prevalence of 45% to less than 20%), with an average 2.4 cm (*p* < 0.005) increase in length at 3 years of age. Exposure to *atole* between 0 and 24 months led to 0.665 US$ (*p* = 0.009) higher hourly wages in adulthood. Authors also demonstrated that a 1-SD improvement in HAZ lead to a 0.78-point (*p* = 0.003) increase in highest grade attained, a 0.28-point (p = 0.003) increase in reading scores, a 0.25-point increase in non-verbal cognitive ability (*p* = 0.002) and a 5-point (p = 0.003) increase in hand strength. Exposure to *atole* between 0 and 12 months caused improvements in reading comprehension scores (6.39 [95%CI: 0.79–11.99]) and in cognitive functioning scores (2.09 [95%CI: 0.79–3.99]). Another successful nutritional supplementation intervention involved a locally produced cereal containing a corn-soya blend with soybean oil and 20 g of protein called *upma* given to children under 6 years and pregnant mothers in South Indian villages from 1987 to 1990. A follow-up analysis of an adolescent cohort (13–18 years old) exposed to *upma* during childhood demonstrated a significant increase in height of the participants (14 mm taller if exposed [*p* = 0.07]) [41]. Nutritional supplementation with *upma* during childhood also resulted in improvements on cardiovascular profiles (20% (*p* = 0.02) lower insulin resistance scores and 3.3% (*p* = 0.008) lower arterial stiffness scores).

Furthermore, a 3-year nutritional supplementation intervention involving multiple micronutrient powders in Pakistan resulted in a 0.2 increase in HAZ score at 6 (*p* < 0.0001) and 18 months (p = 0.02) [42] for the intervention group, compared to a group not receiving nutritional intervention. This intervention resulted in a 3.4-point increase in cognition scores [95%CI: 1.1–5.7], a 5.1-point higher language scores [95%CI: 2.9–7.3] and a 2.9-point higher motor scores [95%CI: 0.5–5.3].

Not all nutritional supplementation interventions successful in stunting reduction resulted in improvement of economic indicators. A 37-week complementary feeding program paired with low iron dose multiple micronutrient powder supplementation resulted in 51% (OR: 0.49, [95%CI: 0.40–0.60]) reduced odds of stunting for the intervention group compared to the control group which received no intervention [28]. However, despite achieving non-significant increases in haemoglobin concentrations, the intervention group developed a higher risk of diarrhea and cold and flu.

#### Economic impact of multisectoral policies successful in reducing stunting

All 4 case studies from sub-Saharan Africa and South America reported positive effects of various multisectoral policies on stunting reduction – ranging from 11 to 20% – over a period of 11 to 14 years (Table 2) [26, 29, 32, 37]. The economic indicator of interest in these studies was childhood mortality.

**Table 2:** Effects on stunting reduction reported in case studies

Country of focus	Intervention period (years)	% Stunting reduction	% Childhood mortality reduction attributed to stunting reduction	AARR for U5MR (%)
Ethiopia	14	20%	44%	5.2%
Niger	11	13%	10%	5.7%
Peru	13	12.5%	N/A	5.3%
Malawi	13	11.1%	8.6%	6.2%

Ethiopia achieved major successes in stunting reduction by focusing on multisectoral, macro-level policies and programmes with a large emphasis on nutrition sensitive and specific programmes such as community-based nutrition programmes to reduce stunting [26]. These efforts resulted in a decrease from 205 deaths/1000 livebirths in 1990 to 64 deaths/1000 livebirths in 2013. Furthermore, Niger’s multisectoral policies and programmes aimed at improving maternal, newborn and child health produced effects in stunting reduction mainly for children aged 24–35 months [32]. Specifically, the Nigerien government implemented 3 National strategies to improve access to health care for women and children, scaled up coverage for high-impact interventions and updated nutrition policies for management of malnutrition which resulted in a 5.1% annual reduction in U5MR in 2012. In Peru, the government implemented policies and programmes aimed at reducing poverty, reforming the health sector, and improving reproductive health, nutrition and maternal and child health [37]. Important strategies were the CCT “Juntos” and the National Strategy for Poverty Reduction and Economic opportunities “CRECER”. The government of Peru was able to decrease U5MR by 58% between 2000 and 2013. Lastly, in Malawi, positive results on stunting reduction were achieved by focusing on high-impact interventions that addressed the main causes of childhood mortality through policies, programmes and funding allocation [29]. Notable actions taken in Malawi were joining the Scaling Up Nutrition movement and creating a National initiative against stunting which focused on nutrition during pregnancy and IYCF with the help of community-based volunteers.

Using the LiST model, investigators were able to determine the proportion of childhood mortality reduction that can be attributed to stunting reduction (Table 2). Observations from 3 of the 4 countries indicated that a reduction in stunting leads to reduced childhood mortality, with countries that achieve higher stunting reduction also estimating a higher attribution to reduced mortality. However, this data was not reported for Peru.

Since only the Niger case study reported an estimate for annual reduction of U5MR, the AARR for U5MR was calculated for each country in order to compare impact of the various multisectoral policies evaluated by the case studies (see Additional file 3: Tables S3-S6 for data used for AARR calculation). The findings demonstrate that all countries experienced positive AARR for U5MR (Table 2). All countries achieved AARR for U5MR higher than 5%, with the highest AARR achieved in Malawi (6.2%). Overall, the AARR estimates for childhood mortality for all 4 countries is aligned with the global target of 4.4% AAAR for U5MR set for the period of 1990–2015 [24]; thus demonstrating a notable speed in progress that can be attributed to multisectoral policies aimed at reducing stunting.

#### Economic impact of cash-based interventions successful in reducing stunting

Conditional cash-based interventions included in this study were implemented in Mexico and Peru and their effects were examined in the included studies following a period of 13 and 20 years respectively. The indicators of interest for CCT studies were human capital indicators.

Two of the 3 studies relaying effects of CCT programmes reported positive effects of the Mexican CCT *Opportunidades* a decade after its implementation in 1998 [39, 40]. *Opportunidades* is based on monthly stipend distributions to households conditional on seeking preventive medical care and school attendance of children past the third grade. An analysis of this intervention showed that a doubling of cash (median increase from 806$ to 1612$) transferred to households was associated with a 0.2 point (*p* < 0.0001) increase in HAZ score and a 10% (p < 0.0001) decrease in stunting. Another analysis of this same CCT demonstrated that exposure to an additional 18 months of the CCT for children of uneducated mothers resulted in a 1.5 cm (*p* = 0.029) increase in HAZ; however, no effect was seen in children of educated mothers. Both studies of *Opportunidades* reported positive effects of the doubling of cash transferred on cognitive outcomes (0.13-point (p < 0.0001) increase in short-term memory and 0.08-point (p = 0.02) increase in visual integration), motor outcomes (0.06-point (*p* = 0.67) increase in motor skills and 1.15-point (*p* = 0.001) increase in endurance), language outcomes (0.18 (*p* < 0.0001) higher language score) and on socioemotional outcomes (0.94-point lower behavioural problems score [p = 0·0024]).

In contrast, a CCT in Peru demonstrated success on stunting reduction 10 years after its implementation in 2005, but was not shown to have positive effects on human capital indicators [38]. *Junto*s is a CCT that provides monthly household stipends conditional on health care visits for children under 5 and pregnant and lactating mothers, as well as school attendance for children between 6 and 14 years old. *Junto*s was successful in increasing HAZ score by 0.43 points (*p* < 0.01) in boys exposed for 2 or more years, but did not report positive effects on the human capital indicators studied (language development and school achievement).

### Quality assessment

#### Cohort studies

For quality assessment of cohort studies, the Newcastle-Ottawa Quality Assessment for Cohort Studies tool was used (Additional file 4: Figure S1). 75% (6/8) of the cohort studies obtained a “good quality” score. However, 2/8 obtained a “fair quality” score because of concern for length and adequacy of the follow up, as well as outcome assessment. Quality rating was given based on specified criteria relating to selection of the participants, classification of exposure and outcome, consideration of confounders and acceptability of follow-up. Overall, the most important bias found was attrition bias (63% of the studies). There was also some concern for selection bias (13%), and exposure and outcome classification (13%).

#### Trials

For the quality assessment of RCTs and the quasi-trial, the Cochrane Risk of Bias tool was used (Additional file 5: Figure S2 and Table S7). There was concern for blinding of participants and personnel in 3 trials (60%), for allocation concealment in 1 trial (20%) and for adequate sequence generation in 1 trial (20%). Furthermore, blinding of the outcome was unclear in 3 studies (60%) and adequate sequence generation and allocation concealment were unclear in 1 study (20%). All studies presented with other biases (Additional file 5: Table S7).

#### Case studies

For quality assessment of case studies, the CEBMa tool [22] was used. All four case studies had focused questions, appropriate study designs, used nationally representative surveys, used methods specific to the authors’ perspective, described data collection methods, presented credible and relevant results, as well as justified conclusions. However, the main concern was for generalizability of the results due to the uniqueness of the context of each country.

## Discussion

Overall, 13 of the 17 included studies had positive effects on stunting, while 11 of the 13 also demonstrated effects on economic indicators. Out of the studies successful in reducing stunting and improving economic indicators, 5 of the 11 evaluated nutritional supplementations, 4 of the 11 evaluated effects of multisectoral policies, and 2 evaluated a CCT.

### Importance of policy and intervention type

Most important results were achieved through multisectoral policies, with 100% success (4 out of 4 studies) on both stunting and childhood mortality [26, 29, 32, 37]. Nutritional supplementation had a 50% success rate, with 5 out of 10 studies evaluating nutritional interventions showing success on both stunting reductions and human capital indicators. It was noted that successful nutrition supplementation involved either protein supplementation or micronutrient supplementation lasting 3 to 7 years [34–36, 41, 42]. Unsuccessful nutrition interventions were micronutrient supplementations implemented over a year or less [28, 33], single-micronutrient supplementations [27, 31] and a 1-year long school feeding program [30]. CCTs reported 67% success (2 out of 3 studies); specifically, the two successful studies evaluated effects of a CCT in Mexico [39, 40], while the unsuccessful CCT was implemented in Peru and failed to demonstrate impact on human capital indicators [38]. The included case studies highlight the importance of multisectoral collaboration. This finding is in line with findings from a previous systematic review that also supports the importance of political commitment and multisectoral interventions, as well as involvement of community members in order to successfully reduce stunting [16]. Thus, although specific interventions are shown to be successful, it is important to keep in mind that the key to having long-lasting impacts on burden of stunting is likely a combination of evidence-based interventions at both the government and community levels.

### Timing of introduction of the intervention

Current research indicates that the first 1000 days of life of a child (from conception to around 3 years of age) is a period of accelerated growth of the brain, body and organs, and emphasizes the importance of this period in determining long-term outcomes [43]. Further research demonstrates that acting during this crucial period also optimizes outcomes [16, 34, 41]. In their study, Hoddinott et al. [34] demonstrate that the intervention was only associated with improvements in wages when the participants had been exposed to *atole* before the age of 3. Moreover, an early childhood and prenatal intervention [41] was shown to have an effect on risk of non-communicable diseases later in life. In the current review, untimely introduction of interventions was noted in the Malawi cohort study where children in their first year of primary school (6–8 years old) were exposed to a school feeding programme for 1 year [30]. This study did not result in any improvements on linear growth of children with one possible explanation being that the intervention was not introduced in the first 1000 days.

### Duration and intensity of the intervention

Some included cohort studies measured outcomes of CCTs in Peru and Mexico. The results of a study on a cohort of children exposed to the CCT *Opportunidades* in Mexico indicated that doubling amounts of cash transfers from approximately 800$ to 1600$ was associated with positive outcomes on stunting reduction, body composition, motor development, cognitive development and language skills [39]. Another cohort study focusing on the same intervention in Mexico indicated that being exposed to the CCT for 18 months longer is beneficial for HAZ scores of children of uneducated mothers, and also has effects on socioemotional outcomes [40]. A very similar CCT in Peru, *Juntos,* has previously been documented as being a key factor in the reduction of stunting in Peru [44]; however, the Andersen et al. study [38] suggests that, unlike *Opportunidades,* this CCT does not have an effect on cognitive outcomes. One explanation could be that the children had not been exposed long enough to this intervention to see results on cognitive outcomes since the sample of children assessed for language and school outcomes were exposed to *Juntos* for under 2 years, while the children assessed in the *Opportunidades* studies had been exposed for 10 years at the time of analysis. Another study potentially reflecting the effect of duration of the intervention on stunting is the Malawi cohort study [30] which only lasted a year and showed no effects on stunting of the children; however, as previously stated, there are other possible reasons that could explain why no effect was seen in this study. Furthermore, micronutrient supplementation studies in Ethiopia and Zambia were implemented for 37 and 52 weeks, respectively [28, 33]. However, such interventions might benefit from multi-year implementation periods in order to have effects on stunting and economic indicators.

### Importance of holistic approaches

Two trials investigated the effects of an oil-based, single-micronutrient supplementation in 6–12 month Ethiopian children and their mothers [27] and 3–9 month old Gambian children [31] for a duration of 12 and 6 months respectively. Both interventions suggest that a single micronutrient supplementation is not an appropriate way of improving childhood morbidity, cognitive delays or growth retardation in children. Furthermore, we included two trials of micronutrient supplementation which were implemented over similar time periods (37 weeks and 52 weeks) [28, 33]. However, only one of those interventions, which combined complementary feeding with micronutrient powders [28], was successful in reducing stunting. Moreover, since stunting is a disorder that affects brain development, it is possible that it is necessary to act beyond nutrition in order to address negative cognitive effects of stunting. A study conducted in Jamaica documented the effect of providing adequate stimulation to children. The authors found that a cohort of stunted children whose parents had been taught how to interact with their children in ways that stimulate cognitive and socioemotional skills, had achieved earnings that were 25% higher than the control group 20 years later, which allowed them to match earnings of a non-stunted comparison group [45]. This study highlights the fact that child development problems are complex and require holistic interventions.

### Strengths and limitations

One of the strengths of the current review is that every study included was assessed for quality. Overall, there was little concern for bias in most studies according to quality scales that were selected based on study design. Furthermore, we calculated AARR for multisectoral policies and were able to link the results to the global target for the AARR for U5MR in order to better illustrate the impact of such policies. This helped strengthen recommendations for multisectoral policies. Furthermore, this document can be used to guide recommendations on how to address stunting. The value that this study adds to existing literature on evidence based approaches to stunting reduction [16] is that it attempts to link select interventions and policies (multisectoral policies, nutritional intervention and cash-based interventions) to stunting reduction and economic outcomes using a wide range of economic indicators in order to fully attempt to capture the potential effect of such policies and interventions on economic growth in low-and-middle-income countries.

One limitation of the current study is the limited amount of information available on the topic. Additionally, none of the studies used advanced modelling or simulation techniques to further investigate the association between stunting and economic development. This limits the generalizability of the results, as there is no quantitative way of associating both outcomes of interest in the studies. It is strongly recommended that future research studies attempt such techniques in order to help improve understanding of the association between stunting and economic outcomes.

Additionally, there are limitations that stem from the articles included. The India cohort study [41] analyzed a cohort of participants that were exposed to both a protein-calorie supplementation and public health interventions; therefore, the outcomes cannot be directly attributed to the nutrition intervention as they may be confounded by competing interventions. Nevertheless, in this review, we report positive results of a protein supplementation in Guatemala, thus the effects of the nutrition intervention cannot be ruled out, despite its possible interaction with other competing interventions. Another limitation is that the case studies all used the LiST tool to assess impact of interventions; however, the LiST tool does not take all variables into account and does not consider important aspects such as economic growth which could also have an impact on stunting reduction. Lastly, it is difficult to state whether or not these interventions can be replicated because they each took place in unique contexts and different countries or regions may not have access to the same resources and the socio-political environment might differ.

Furthermore, a meta-analysis was not possible due to incoherence in the measures used to assess economic indicators across studies. Only the 4 case studies presented results that were cohesive enough for a meta-analysis; however, the sample was considered too small to produce meaningful results. Standardization of economic indicators should therefore be prioritized to allow for future meta-analyses.

Lastly, the studies included in this review were conducted in Latin America, sub-Saharan Africa and South Asia and direct inference cannot be made to other regions that lack evidence and that are struggling with high stunting rates. Taking the example of the Middle East, the most recent figures report that stunting affects approximately 15.1 million children in the region [8]. Given the high reported caseload, it would be of added value to advocate for piloting successful interventions and policies identified in our review and promoting research studies in this region. In summary, our findings as well as those from other reviews that discuss cost-effectiveness of interventions [9, 10] can help leverage discussions with decision-makers to mobilize resources in favour of stunting reduction.

## Conclusion

In conclusion, an array of interventions and policies are being piloted and researched in many parts of the world with the aim of accelerating the fight against chronic malnutrition and ultimately reach the goal of reducing stunting by 40% by 2025. The findings of this review suggest that investment in chronic malnutrition through multisectoral policies, multi-year nutritional supplementation (protein or multiple micronutrient supplementation) and possibly CCTs can have a long-term impact on economic development of LMICs. The lack of standardization of economic indicators has been noted in this review and should be addressed in future studies to allow for meta-analyses and the strengthening of recommendations. Furthermore, the limited number of articles in this review demonstrates the need for additional evidence on this topic to help generate political commitment to stunting reduction. In particular, more evidence is needed from regions that were not represented in this review in order to gain more insights into how the interventions and policies identified as successful in this study function in the specific contexts of those regions. Governments must be reminded that evidence-based action is key and that reducing stunting will ultimately reward countries with considerable economic returns. Lastly, it is important to bear in mind that every child that is allowed to become stunted is being deprived of important and necessary resources which are crucial in supporting children in achieving their full potential and having successful futures.

## Supplementary information

**Additional file 1:** **Table S1.** Description of terminology for economic indicators (PDF 29 kb)

**Additional file 2:** **Table S2.** Search terms for PubMed. (PDF 41 kb)

**Additional file 3:** **Tables S3–S6.** WorldBank data (2000–2017) used to calculate AARR for Ethiopia, Malawi, Peru and Niger. (PDF 56 kb)

**Additional file 4:** **Figure S1.** Quality assessment of cohort studies. (PDF 59 kb)

**Additional file 5:** **Figure S2 and Table S7.** Quality assessment of trials. (PDF 67 kb)

## Data Availability

The datasets supporting the conclusions of this article are included within the article (and its Additional files).

## Abbreviations

AARR: Average Annual Rate of Reduction
CCT: Conditional cash transfers
CEMBa: Center for Evidence-Based Management
GDP: Gross Domestic Product
HAZ: Height-for-age
IYCF: Infant and Young Child Feeding
LMICs: Low- and middle-income countries
MUAC: Mid-upper-arm-circumference
OECD: Organization for Economic Co-operation and Development
PRISMA: Preferred Reporting Items for Systematic Reviews and Meta-Analyses
RCT: Randomized Control Trial
U5MR: Under 5 mortality rate
WHA: World Health Assembly
