# Supplementary material for: Do nutrition and cash-based interventions and policies aimed at reducing stunting have an impact on economic development of low-and-middle-income countries? A systematic review
Source: BMC Public Health. 2019 Oct 30;19:1419. doi: 10.1186/s12889-019-7677-1 (PMC6820910; doi:10.1186/s12889-019-7677-1)
Supplement: Supplementary file 1 — Additional file 1: Table S1. Description of terminology for economic indicators (PDF 29 kb) [file 12889_2019_7677_MOESM1_ESM.pdf]

Additional file 1. Supplementary table 1

Supplementary table 1– Description of terminology for economic indicators.

**Economic indicator**		**Specific terminology**
**Childhood mortality**		Under 5 mortality rate (U5MR); child morbidity, child health
**Human capital indicators**		
	Cognitive health	*language development; school achievement; cognitive development; motor development; socioemotional development; cognitive ability; reading comprehension; cognitive functioning; schooling-related outcomes; behavioural problems*
	Economic outcomes	*annual income hours worked; average hourly wages; labor market outcomes*
	Chronic disease	*adult health outcomes; cardiovascular health*
