# Supplementary material for: Do nutrition and cash-based interventions and policies aimed at reducing stunting have an impact on economic development of low-and-middle-income countries? A systematic review
Source: BMC Public Health. 2019 Oct 30;19:1419. doi: 10.1186/s12889-019-7677-1 (PMC6820910; doi:10.1186/s12889-019-7677-1)
Supplement: Supplementary file 2 — Additional file 2: Table S2. Search terms for PubMed. (PDF 41 kb) [file 12889_2019_7677_MOESM2_ESM.pdf]

Additional file 2. Supplementary table 2

Supplementary table 2– Search terms for PubMed

#1 nutrition policy/ intervention	“nutrition”[tw] OR “food”[tw] OR food strateg*[tw] OR “policy” [MeSH] OR polic*[tw] OR “nutrition policy”[MeSH] OR nutrition* polic*[tw] OR nutrition* guideline*[tw] OR diet* guideline*[tw] OR food polic*[tw] OR “nutrition intervention”[tw] OR food polic* [tw] OR “stunting policy”[tw] OR “malnutrition policy”[tw] OR “WaSH” OR “water, sanitation and hygiene“[tw] OR “water”[tw] OR “sanitation”[tw] OR “hygiene”[tw] OR “growth monitoring and promotion”[tw] OR cash based intervention*[tw] “conditional cash transfers”[tw] OR nutrition* strategy[tw] OR “nutrition services”[tw] OR “services”[tw] OR deworm*[tw] OR de-worm*[tw] OR breastfeed* program[tw] OR exclusive breastfeed*[tw] OR “baby-friendly initiative”[tw] OR food and nutrition polic*[tw] OR nutrition* intervention*[tw] OR “nutrition strategy”[tw] OR “national nutrition strategy”[tw] OR “public programs on child health”[tw] OR “nutrition service*”[tw] OR “growth monitoring and promotion program”[tw] OR “deworming program” [tw] OR “de-worming program”[tw] OR deworming intervention* [tw] OR de-worming intervention*[tw] OR breastfeed* program*[tw] OR “complementary feeding”[tw] OR nutrition* supplementation[tw] OR “supplementation”[tw] OR complementary food*[tw] OR “complementary”[tw] OR “food fortification”[tw] OR fortified food*[tw] OR “promotion” OR feeding program*[tw] OR evidence-based intervention*[tw] OR evidence-based program*[tw] OR evidence-based polic*[tw] OR “therapeutic feeding”[tw] OR “nutrition program*” OR “feeding”[tw] OR “large-scale”[tw] OR “infant and young child feeding”[tw] OR “IYCF”[tw] OR “CMAM”[tw] OR “community-based management of acute malnutrition”[tw] OR “community management”[tw] OR community[tw] OR “mobile intervention”[tw] OR cash[tw] OR “early childhood”[tw]
#2 stunting	stunt*[tw] OR “growth disorders”[MeSH] OR “stunted growth“[tw] OR “poor linear growth”[tw] OR “low height-for-age”[tw] OR “malnutrition”[MeSH] OR “malnutrition”[tw] OR nutritional deficienc*[tw] OR malnourish*[tw] OR severe stunt*[tw] OR severely stunt*[tw] OR stunted child*[tw] food insufficienc*[tw] OR “food insecurity”[tw] OR “growth faltering”[tw] OR child* stunting[tw] OR “chronic malnutrition”[tw] OR “poor growth outcomes”[tw] OR “growth retardation”[tw] OR “malnourished”[tw] OR “stature”[tw] OR “height”[tw] OR “first 1000 days”[tw] OR 1000 day* window[tw] OR “first 1,000 days”[tw] OR 1,000 day* window[tw] OR “ inadequate nutrient intake”[tw] OR “stunted brain”[tw] OR undernutrition [tw] OR under-nutrition[tw] OR undernourish*[tw] OR “height-for-age”[tw] OR “length-for-age”[tw]
#3 economic	social[tw] OR economic[tw] OR socioeconomic impact*[tw] OR “socioeconomic effect”[tw] OR socioeconomic consequence*[tw] OR impact[tw] OR consequence OR “socioeconomic status” [tw] OR “social class”[MeSH] OR social class*[tw] OR “ Economic development”[MeSH] OR economic development*[tw] OR “ socioeconomic disadvantage”[tw] OR “vulnerable populations”[MeSH] OR vulnerable population*[tw] OR vulnerable individual*[tw] OR disadvantage*[tw] OR underserved population*[tw] OR “socioeconomic disparities” [tw] OR socio-economic*[tw] OR socioeconomic*[tw] OR economic*[tw] OR wage* [tw] OR salar*[tw] OR development* [tw] OR “economic growth”[tw] OR “income”[tw] OR “salaries and fringe benefits”[MeSH] OR earn*[tw] OR hourly wage*[tw] OR hourly-wage*[tw] OR health[MeSH] OR health*[tw] “health care costs”[MeSH] OR health care cost*[tw] OR health cost*[tw] OR healthcare cost*[tw] OR medical care cost*[tw] OR treatment cost*[tw] OR “social development”[tw] OR “social change”[MeSH] OR social change*[tw] OR social impact*[tw] OR “economic growth”[MeSH] OR “ socioeconomic factor”[MeSH] OR socioeconomic factor*[tw] OR “socio-economic factor” OR inequalit*[tw] OR living standard*[tw] OR sustainable development

	goal*[tw] OR millennium development goal*[tw] OR “cost-benefit”[tw] OR “cost-benefit analysis”[tw] OR “cost-effectiveness analysis”[tw] OR “cost-effective”[tw] OR “cost-effectiveness” OR “cost-efficiency”[tw] OR “cost-efficiency analysis”[tw] OR “cost-efficient” OR “years in school”[tw] OR “highest grade achieved”[tw] OR “level of education”[tw] OR education[MeSH] OR education[tw] OR literacy[tw] OR literate[tw] OR illiterate[tw] OR “literate population”[tw] OR “economic productivity”[tw] OR “human development”[tw] OR “lost labor productivity”[tw] OR “labor productivity”[tw] OR productivity[tw] OR mortality[tw] OR “quality of life”[tw] OR “expected earning*”[tw] OR economic cost*[tw] OR economic benefit*[tw] OR benefit*[tw] OR cost*[tw] OR cognitive impairment*[tw] OR “labor market outcome”[tw] OR “labor market”[tw] OR work outcome*[tw] OR schooling outcome*[tw] OR “grade attainment”[tw] OR “grade achieved”[tw] OR “years of schooling”[tw] OR “social skills”[tw] OR “cognitive ability”[tw] OR “cognitive development”[tw]
#4 low- and middle-income countries	“low- and middle- income”[tw] OR low and middle income countr* [tw] middle-income countr* [tw] OR low-income countr*[tw] OR “developing countries”[MeSH] OR developing countr*[tw] OR least developed countr*[tw] OR less-developed countr*[tw] OR less developed countr*[tw] OR “under-developed”[tw] OR under-developed countr*[tw] OR third-world countr*[tw] OR OR third world countr*[tw] OR “third world”[tw] OR “third-world”[tw] OR developing nation*[tw] OR less-developed nation*[tw] OR less developed nation*[tw] OR “low-income”[tw] OR “middle-income”[tw] NOT (high-income countr*[tw] OR high income countr* OR developed countr*[tw])
#5 high-income countries	“high-income countr*” [tiab] OR “high income countr*”[tiab] OR “developed countr*”[tiab] OR “USA”[tiab] OR “united states”[tiab] OR "U.S."[tiab] OR “north America”[tiab] OR “American”[tiab] OR obesity[tiab] OR diabetes[tiab] OR “climate change”[tiab] OR obese[tiab] OR "AIDS"[tiab] OR "HIV"[tiab] OR vaccin*[tiab] OR polio[tiab]
#5	#1 AND #2 AND #3 AND #4 NOT #5
