# Supplementary material for: Do nutrition and cash-based interventions and policies aimed at reducing stunting have an impact on economic development of low-and-middle-income countries? A systematic review
Source: BMC Public Health. 2019 Oct 30;19:1419. doi: 10.1186/s12889-019-7677-1 (PMC6820910; doi:10.1186/s12889-019-7677-1)
Supplement: Supplementary file 3 — Additional file 3: Tables S3–S6. WorldBank data (2000–2017) used to calculate AARR for Ethiopia, Malawi, Peru and Niger. (PDF 56 kb) [file 12889_2019_7677_MOESM3_ESM.pdf]

Additional file 3. Supplementary tables 3-6

Supplementary table 3– WorldBank estimates for Ethiopia U5MR (2000-2017)

Year	U5MR Prevalence
2000	142.6
2002	130.4
2004	117.4
2006	104.9
2008	93.5
2010	83.7
2011	79.2
2013	71.2
2016	61.2
2017	58.5
Calculated AARR	5.20%

Supplementary table 4– WorldBank estimates for Malawi U5MR (2000-2017)

Year	U5MR Prevalence
2000	171.9
2002	146.4
2004	122.6
2006	107.7
2008	97.7
2010	88.6
2012	75.9
2014	67.5
2016	61.2
2017	55.4
Calculated AARR	6.20%

Supplementary table 5– WorldBank estimates for Peru U5MR (2000-2017)

Year	U5MR Prevalence
2000	38.7
2002	33.1
2004	28.7
2006	25.2
2008	22.5
2010	20.3
2012	18.5
2014	16.9
2016	15.5
2017	15
Calculated AARR	5.30%

Supplementary table 6– WorldBank estimates for Niger U5MR (2000-2017)

Year	U5MR Prevalence
2000	223.7
2002	203.1
2004	180.4
2006	158.4
2008	139.6
2010	123.6
2012	109.9
2014	98.6
2016	88.7
2017	84.5
Calculated AARR	5.70%
