# Supplementary material for: Do nutrition and cash-based interventions and policies aimed at reducing stunting have an impact on economic development of low-and-middle-income countries? A systematic review
Source: BMC Public Health. 2019 Oct 30;19:1419. doi: 10.1186/s12889-019-7677-1 (PMC6820910; doi:10.1186/s12889-019-7677-1)

Additional File 4. Supplementary figure 1

Supplementary Figure 1 – Quality assessment of cohort studies

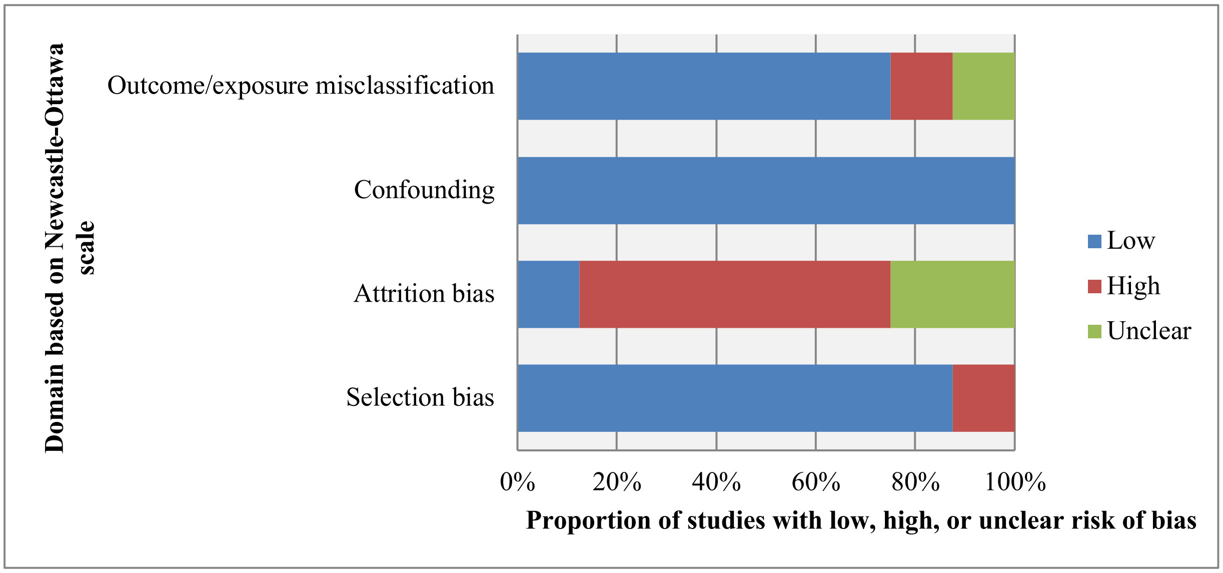

Supplement: Supplementary file 4 — Additional file 4: Figure S1. Quality assessment of cohort studies. (PDF 59 kb) [file 12889_2019_7677_MOESM4_ESM.pdf]
