# Supplementary material for: Do nutrition and cash-based interventions and policies aimed at reducing stunting have an impact on economic development of low-and-middle-income countries? A systematic review
Source: BMC Public Health. 2019 Oct 30;19:1419. doi: 10.1186/s12889-019-7677-1 (PMC6820910; doi:10.1186/s12889-019-7677-1)
Supplement: Supplementary file 5 — Additional file 5: Figure S2 and Table S7. Quality assessment of trials. (PDF 67 kb) [file 12889_2019_7677_MOESM5_ESM.pdf]

Additional File 5. Supplementary figure 2 and supplementary table 7

Supplementary Figure 2 – Quality assessment of trials

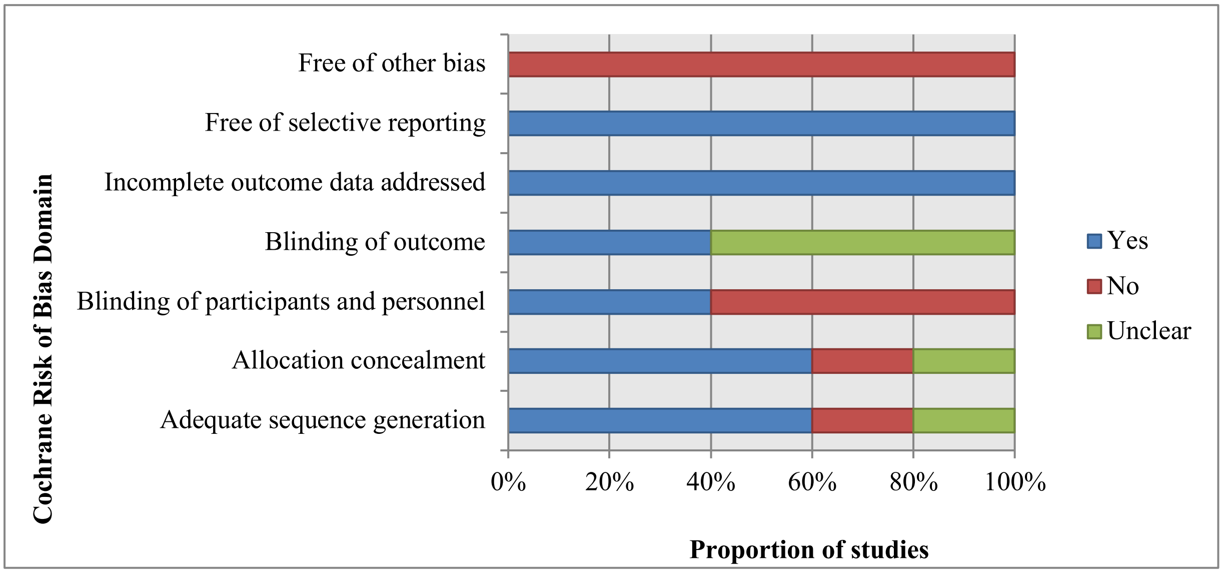

Supplementary Table 7 – Other biases in trials

**Type of bias**	**Frequency of appearance in trials**	**%**
sampling bias	2	29%
selection bias	1	14%
Self-reported outcomes (desirability bias)	2	29%
recall bias	2	29%
**Total**	**7**	**100%**
